# Effect of Aging Treatment on the Precipitation Behavior of a Novel Al-Cu-Zr Cast Alloy

**DOI:** 10.3390/ma15228163

**Published:** 2022-11-17

**Authors:** Wu Wei, Rui Zuo, Da Xue, Shengping Wen, Yang Wu, Wei Shi, Xiaorong Zhou, Hui Huang, Xiaolan Wu, Kunyuan Gao, Li Rong, Zuoren Nie

**Affiliations:** 1 Key Laboratory of Advanced Functional Materials, Education Ministry of China, Beijing University of Technology, Beijing 100124, China; 2 Institute of Corrosion Science and Technology, Guangzhou 510530, China; 3 Department of Materials, The University of Manchester, Manchester M13 9PL, UK

**Keywords:** Al-Cu-Zr cast alloy, two-step aging, θ’ phases, Al_3_Zr phases, precipitation behavior

## Abstract

A novel Al-Cu-Zr alloy is designed in this paper, which provides a method for further improving the strength of Al-Cu alloys. In this paper, the addition of the micro-alloying element Zr in Al-Cu alloy was studied. The effect of aging treatment on the mechanical properties and precipitation behavior of the alloy was studied. With the addition of Zr, Al_3_Zr phases were formed in the alloy, which acts as obstacles to dislocation motion. In addition, Al_3_Zr phases can be used as the nucleation site of θ′ phases to promote precipitation. All this can improve the strength of Al-Cu alloys. After one-step aging, corresponding to the highest hardness, the largest amount of θ′ phases were observed in the alloy matrix. By contrast, after two-step aging, the θ′ phases were finer, and a large amount of Guinier–Preston (GP) zones formed during the pre-aging step, which were transformed into denser and finer θ′ phases in the secondary aging step. After the same solution treatment (540 °C/12 h), undergoing 120 °C/4 h + 175 °C/10 h two-step aging, the ultimate tensile strength, yield strength, and elongation of the Al-Cu-Zr alloy were 398.7 MPa, 313.3 MPa, and 7.9%, respectively.

## 1. Introduction

In recent years, with the rapid development of industry, researchers have devoted increasing attention to light structural materials which have higher specific strength. Among them, Al-Cu alloys are widely used in the automotive industry and aerospace field because of their high specific strength and good fracture toughness [1,2]. As cast alloys, Al-Cu alloys are not subject to any deformation processes, which can reduce production costs in the manufacturing process. Its high strength is obtained by precipitation hardening. The precipitation sequence of Al-Cu alloy is supersaturated solid solution (SSSS) → Guinier–Preston (GP zones) → theta-double prime (θ″) precipitate → theta-prime (θ′) precipitate → theta (θ) precipitate [3]. These precipitation phases are affected by the chemical composition and temperature [4,5]. The θ′ phase plays a major strengthening role in Al-Cu alloys [3,6,7]. In general, the θ′ phase is considered to be disc-like and semi-coherent with the α-Al matrix. When the aging temperature is high, the θ’ phase transfers to the θ phase and their compositions approach Al_2_Cu. The θ phase has a little strengthening effect on the alloy [8].

In order to further improve the mechanical properties of the Al-Cu alloy and not decreased the elongation of the alloy, in recent years, scholars have proposed to improve the mechanical properties of Al-Cu alloys through multi-step aging [9,10]. Compared with one-step aging, multi-step aging can obtain finer and more dispersed precipitation phases, which can improve the mechanical properties of Al-Cu alloys [11,12]. Elgallad et al. [4] studied the effect of multi-step aging strengthening on the properties of the AA2219 direct chill alloy and found that compared with one-step aging, two-step aging at 120 °C/36 h + 190 °C/8 h increased the precipitation in GP zones, and the θ′ phase was significantly smaller than one-step aging. Wang et al. [13] studied the effect of two-step aging on the mechanical properties and microstructure of the cast Al-4.5Cu-3.5Zn-0.5Mg alloy. In the initial aging stage, small Cu-rich Guinier–Preston (GP) zones and Mg-rich clusters (GPI zones) are formed, which promotes the formation of finer and denser θ′ phases and Ω phases. Lumley [14] adopted the T6I4 interrupted aging to improve the mechanical properties of an Al-Cu-Mg-Mn alloy by first under-aging at T6, water quenching, then aging at room temperature or slightly above room temperature. Through T6I4 interrupted aging, finer and denser Ω phases are precipitated, which causes a great improvement in the hardness and strength of the alloy.

At present, there are few studies on the addition of Zr to Al-Cu alloys, and the effect mechanism of Zr on the precipitation behavior of Al-Cu alloys is not clear. Zr can not only reduce the production cost [15], but also improve the fluidity of liquid alloy, and react with Al to generate the Al_3_Zr phase, refining the grain and improving the mechanical properties of the alloy [16]. The solubility of Zr in α-Al is 0.083 at.%, and the Al_3_Zr phase with L_12_ structure is generated in the aluminum alloy, which is a face-centered cubic structure with a 0.75% mismatch with α-Al [17]. Therefore, the formed Al_3_Zr phase can act as a heterogeneous nucleation site to produce non-uniform nucleation to refine the grains and improve the thermal stability of the alloy. Shower et al. [18] and Populaski et al. [19] found that Zr would preferentially segregate to the Al-substrate/θ′ phase interface and was able to reduce the coarsening kinetics of the θ′ phase, indirectly achieving the effect of precipitation strengthening. In this experiment, a new Al-Cu-Zr alloy was designed to study the effect of aging strengthening on the mechanical properties and precipitation behavior of the Al-Cu-Zr alloy. To find the best aging process route, the role of Zr in the aging process of Al-Cu alloys was studied in detail.

## 2. Materials and Methods

**Figure 1:**
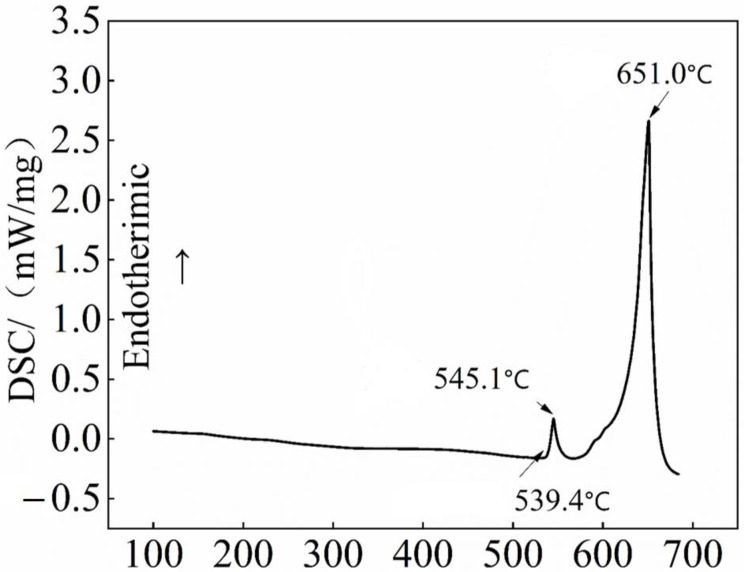
DSC curves of the cast Al-Cu-Zr alloys.

The material used in this experiment is an Al-Cu-Zr alloy, it was obtained by casting commercial pure Al, Mg, and intermediate alloys Al-50Cu, Al-10Mn, Al-10Zr, and grain refining agents Al-6Er and Al-5% Ti-1%B. This is shown in Table 1. It was melted using a laboratory resistance furnace with a melting temperature of 720 °C, to perform metal type casting. The optimal solid solution temperature was determined according to DSC-540 °C in Figure 1. The solid solution treatment was at 540 °C for different times, and the highest hardness was found at 12 h. So the heat treatments involved were solution treated (540 °C/12 h) → water quenching → various aging treatments, as shown in Table 2. To determine the optimum aging time, aging treatment at 155 to 195 °C with an interval of 10 °C up to 48 h was carried out in an air circulation furnace (KSL-1000X, China).

Vickers microhardness (HV) measurements were by HXD-1000TM/LCD Vickers hardness tester, at a load of 100 gf applied for 10 s, the data reported here represent the average of at least nine times. The alloy specimen was optically observed using an OLYMPUS PMG3 optical microscope. The samples were etched by using a Keller reagent consisting of 95% H_2_O (vol.%), 2.5% HNO_3_ (vol.%), 1.5% (vol.%) HCl, and 1.0% (vol.%) HF. The microstructures of the as-cast and solution-treated samples were examined using a scanning electron microscope (FEI QUANTA FEG 650) equipped with an energy-dispersive spectrometer (EDS). The samples used for the transmission electron microscopy (JEM-2100F) observations were manually thinned to 70 μm, then small discs with a diameter of 3 mm were punched out from the samples, and at last electropolished to perforation with a double spray in a solution of 30% CH_3_OH and 70% HNO_3_ at −25 °C.

The alloy specimen was used to stretch test with GB228−2010. In order to ensure the reproducibility of the tensile results, three tensile tests were performed for every specimen.

## 3. Results

### 3.1. As-Cast and Solution-Treated Microstructures

**Figure 2:**
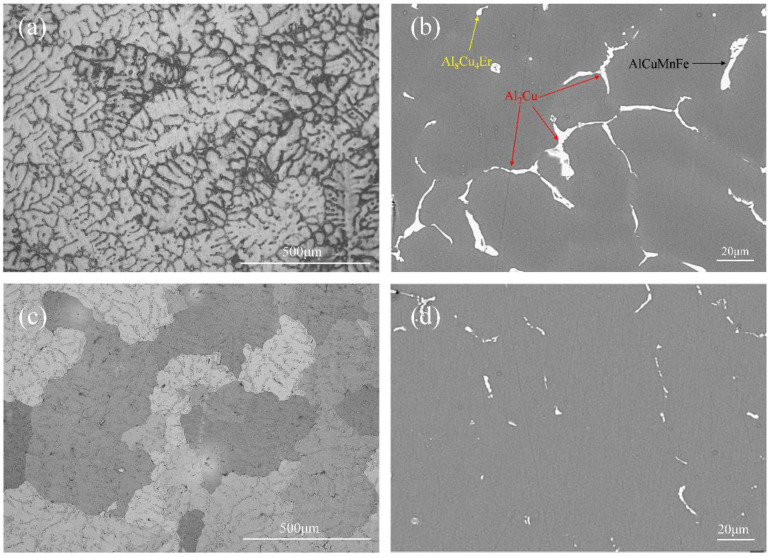
Optical microstructures and backscattered electron SEM images of Al-Cu-Zr alloy: (**a**) OM image of cast alloy, (**b**) BSE image of cast alloy, (**c**) OM image of solid solution, and (**d**) BSE image of solid solution.

The Al-Cu-Zr cast alloy was initially investigated. Figure 1 shows the alloy optical microstructure and backscattered electron image of the as-cast and solution treated at 540 °C for 12 h. Figure 2a shows the optical microstructure, and a large amount of dendrite segregation can be observed. The main cause of this phenomenon is non-equilibrium solidification. The backscattered electron image shown in Figure 2b displays three major intermetallic phases: the Al_2_Cu phase, the Al_8_Cu_4_Er phase, and the Al-Cu-Mn-Fe impurity phase. Al_2_Cu is the main reinforcement phase, after solid solution treatment, it can be dissolved into the α-Al matrix. In contrast, the Al_8_Cu_4_Er and Al-Cu-Mn-Fe impurity phases are difficult to dissolve at this temperature, and both adversely affect the mechanical properties of the alloy. Figure 2c shows the alloy microstructure after the solution was treated at 540 °C for 12 h, and dendrite segregation has been largely eliminated compared to cast structures (reduced from 5.96% to 1.42%). The backscattered electron image shown in Figure 2d shows that most of the Al_2_Cu precipitation phases have been reintegrated into the α-Al except for small particles; residual phases are Al_8_Cu_4_Er and Al-Cu-Mn-Fe impurity phases.

### 3.2. One-Step Aging

**Figure 3:**
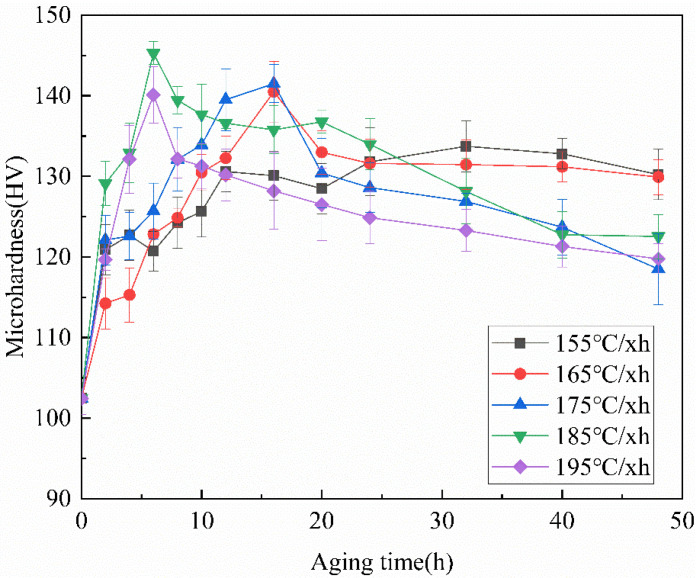
Microhardness curves of the Al-Cu-Zr alloy studied at different temperatures of one-step aging.

Figure 3 shows the hardness curves after aging at different temperatures. It can be seen that within the measured temperature range, the hardness of samples after aging treatment shows a trend of rising first and then falling, and the time required to reach the peak hardness is gradually shortened with the increase of aging temperature. After aging at 155 °C for 32 h, the hardness reached the peak of 133.7 HV. The peak hardness was reached at 165 °C and 175 °C after holding for 16 h, and the hardness values were 140.5 HV and 141.5 HV, respectively. When the aging temperature continues to rise, whether it is 185 °C or 195 °C, it only needs to hold for 6 h to reach peak aging, greatly shortening the aging time. The hardness of peak aging at 185 °C is higher than that of other temperatures, which is 145.3 HV. It can be determined that aging at 185 °C for 6 h is the best one-step aging state of the Al-Cu-Zr alloy. The peak hardness is 140.1 HV at 195 °C. Tensile tests were carried out on all alloys in peak aging states, and ultimate tensile strength (UTS), yield strength (YS), and elongation (EL) to failure were obtained, the results are shown in Table 3. The best mechanical properties are achieved under peak aging at 185 °C, UTS and YS are higher than those under peak aging at other temperatures, which is consistent with the results of the hardness curve. Compared with the as-cast alloy, its UTS, YS, and EL increased by 66.5%, 66.6%, and 43.8%, respectively. This is related to the precipitation of the θ′ phase during aging.

**Figure 4:**
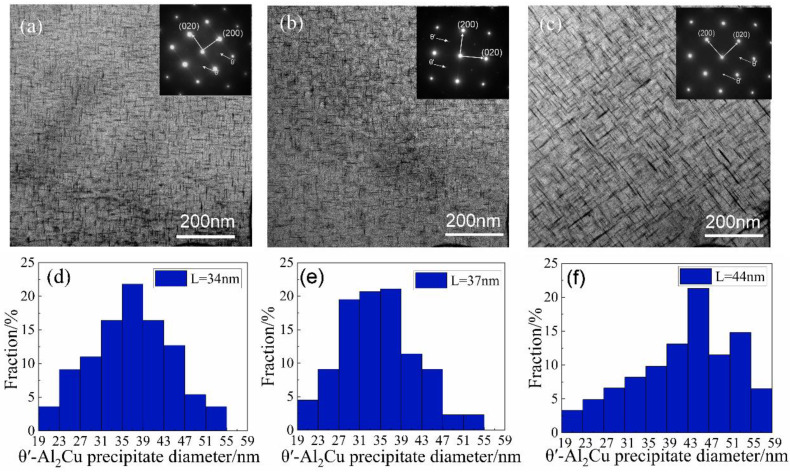
TEM bright-field images of the alloy after one-step peak aging at different temperatures and the corresponding length size distribution histograms of θ′ phases: (**a**) 165 °C for 16 h; (**b**) 175 °C for 16 h; (**c**) 185 °C for 6 h (the corresponding length size distribution histograms of the θ′ plates are placed below the images); (**d**) θ′ phases size after aging at 165 °C/16 h; (**e**) θ′ phases size after aging at 175 °C/16 h; (**f**) θ′ phases size after aging at 185 °C/6 h.

The evolution of the mechanical properties after applying the one-step aging treatment will be explained by characterizing the precipitate microstructure using TEM observations. Figure 4 shows the size and distribution of precipitated phases in the Al-Cu-Zr alloy after single peak aging at 165, 175, and 185 °C. The corresponding selected area electron diffraction (SAED) pattern shows that the precipitates after different aging treatments are θ′ phases. These θ′ phases are uniformly distributed in the alloy as plates; the aging temperature and time determine the nucleation and growth process of these precipitated phases. Figure 4a,b shows TEM images of the alloy after aging at 165 and 175 °C for 16 h. It can be seen that the θ′ phase number density does not change significantly after the aging treatment at 175 °C compared with 165 °C, but remains in a stable state. Under these two states, the average length of θ′ precipitated phases changes from 34 nm to 37 nm, the average width changes from 4.0 nm to 4.1 nm, and the sizes of precipitated phases have little change. This explains the fact that the mechanical properties of the alloys in the two states shown in Table 3 are basically the same. Figure 4c shows the size and distribution of θ′ phases in the Al-Cu-Zr alloy after aging at 185 °C for 6 h. The number of θ′ phases increased significantly after peak aging at 185 °C compared with the first two conditions. Although the alloy in this state is only held for 6 h, the average length of the precipitated phase is much larger than the size of the precipitated phase held at 165 °C and 175 °C, reaching 44 nm, increasing by more than 15%. The average width was 4.5 nm, an increase of 10%. This shows that the effect of aging temperature on precipitate is much greater than that of holding time in one-step aging. According to Table 3, the mechanical properties of the alloy after peak aging at 185 °C are better than those of the other two states, indicating that the influence of the number of precipitated phases on the mechanical properties of the alloy is greater than that of the size of the precipitated phase.

### 3.3. Two-Step Aging

**Figure 5:**
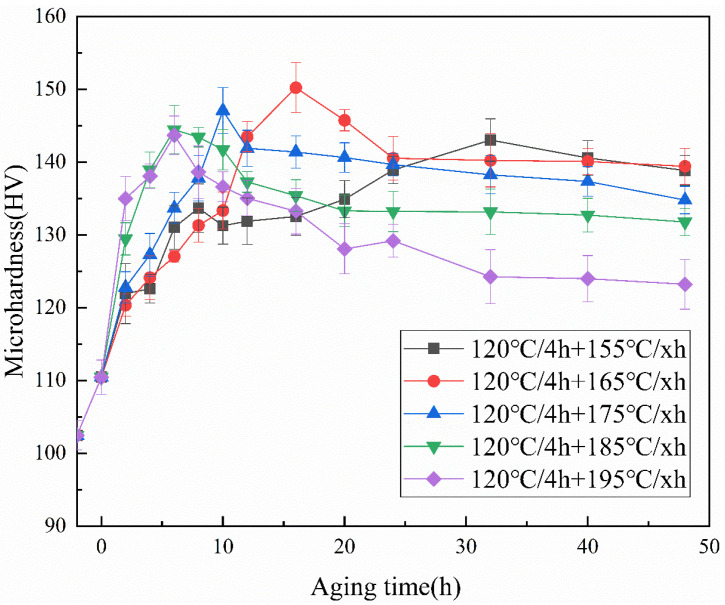
The microhardness curves of the Al-Cu-Zr alloy studied at different temperatures of two-step aging.

Compared with one-step aging, two-step aging adds a pre-aging stage. This increases the number of GP zones. Based on the work of Lorimer and Nicholson, GP zones grow at a lower aging temperature. With the increase in aging temperature, these GP zones can continue to grow, and at the same time, these GP zones and stable clusters can be used as nucleation sites in the second-step aging process to accelerate the aging process [20]. In order to determine the best temperature and time for secondary aging, different aging times ranging from 0 to 48 h were examined at temperatures of 155~195 °C. Figure 5 is the microhardness curve of the alloy after different aging treatments. After two-step aging, the hardness of the alloy still shows a trend of rising first and then decreasing with the increase of aging temperature, and the peak hardness is greatly improved compared with one-step aging. At 155 °C, the peak hardness of the alloy reaches 143 HV, which is an increase of 7.1% compared with one-step aging. When the aging temperature is 165 °C, the peak hardness of the alloy is 150.2 HV, an increase of 7.0% compared with one-step aging, and the peak hardness of the alloy at this temperature is higher than other temperatures, so it can be determined as the best state of two-step aging. When the aging temperature rises to 175 °C, the hardness of the alloy under peak aging is 147.1 HV, which is an increase of 4% compared with one-step aging and is a slight decrease compared with the previous. When the aging temperature continues to rise to 185 °C and 195 °C, the peak hardness of the alloy is 144.5 HV and 143.7 HV, respectively, which is not a significant increase compared with one-step aging. This situation may occur because although the number of precipitated phases increases, the precipitated phase is coarsening due to the higher temperature and the increase in overall aging time. At the same time, compared with one-step aging, it can be found that when the temperature is lower, due to the pre-aging, the time required for the alloy to reach peak aging is reduced, but this situation does not occur in the high-temperature aging step, because when the temperature is higher, the higher aging temperature can provide high enough energy for atomic activities. Therefore, there is no significant effect on the peak aging rate of the alloy during high-temperature aging.

The mechanical properties of the Al-Cu-Zr alloy under different heat treatment conditions are listed in Table 3. The mechanical properties are the best at 165 °C peak aging, which is consistent with the hardness curve. Its Uts is 413 MPa, Ys is 322.6 MPa, and El is 7.6%, which are 76.7%, 86.2%, and 137.5% higher than those of as-cast alloy, respectively. As the aging temperature increased from 155 to 165, 175, 185, and 195 °C, UTS increased from 373 to 413, 398.7, 384.3, and 374.4 MPa, YS increased from 295.7 to 322.6, 313.3, 307.3, and 304.6 MPa, while the elongation changed from 7.4% to 7.6%, 7.9%, 7.8%, and 6.7%.

**Figure 6:**
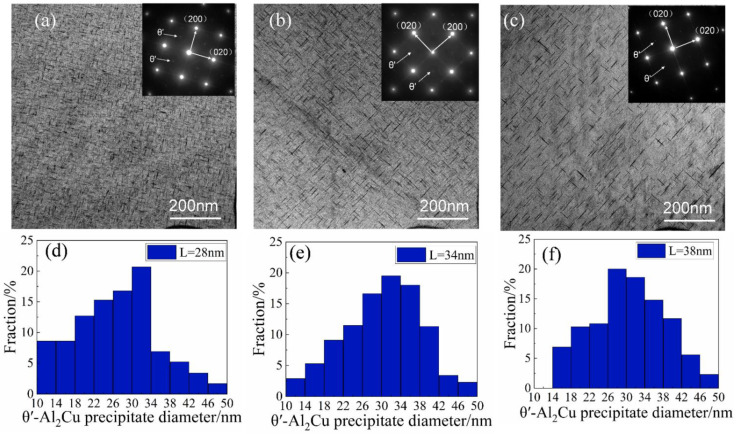
TEM bright-field images of the alloy after different two-step aging temperatures and the corresponding length size distribution histograms of θ′ phases (the pre-aging all are 120 °C/4 h): (**a**) 165 °C for 16 h; (**b**) 175 °C for 10 h; (**c**) 185 °C for 6 h (the corresponding length size distribution histograms of the θ′ phases are placed below the images). (**d**) θ′ phases size after aging at 120 °C/4 h + 165 °C/16 h; (**e**) θ′ phases size after aging at 120 °C/4 h + 175 °C/10 h; (**f**) θ′ phases size after aging at120 °C/4 h + 175 °C/10 h.

The evolution of the mechanical properties after applying the two-step aging treatment will be explained by characterizing the precipitate microstructure using TEM observations. Figure 6 shows TEM bright-field images of peak aging at 165 °C, 175 °C, and 185 °C as well as selected area electron diffraction (SAED) and statistical histograms of precipitated phase size. As shown in Figure 6, the average precipitate size appeared to increase with increasing aging temperature, while the morphology did not change significantly. The corresponding selected area electron diffraction (SAED) pattern recorded at the [001] α zone axis had characteristic θ′ spots, indicating that these precipitates are θ′ phase particles. It indicates that the type of precipitated phase has no change with the aging regime. Even when the aging temperature increases, the precipitated phases are still the θ′ phase and do not change into the θ phase. The decline of mechanical properties is not caused by the phase’s transformation. According to Figure 6a, after pre-aging at 120 °C/4 h and peak aging at 165 °C, the quantity of θ′ phases in the alloy increases significantly, which is higher than that after 175 °C and 185 °C secondary aging, and the size of precipitated phase is smaller. As can be seen from Figure 6d, the average length and width of the θ′ phase at this time are 28 nm and 3.8 nm, respectively, the length and width are smaller than the other two cases. The relatively large number density and small size of precipitation cause it to possess good mechanical properties, which is consistent with the results in Table 3. When the temperature of the secondary aging increases to 175 °C and 185 °C, it can be seen from Figure 6b,c that the number density of the θ′ phase is significantly lower, and coarsening of the θ′ phase occurs to different degrees due to the increase of temperature. The average diameters of the θ′ phase are 34 nm and 38 nm, and the average widths are 4 nm and 4.2 nm, respectively. Compared with the two-step peak aging at 165 °C, the size of the precipitated phase increased significantly. Based on these observations, in Figure 6d,e we show increments in average size are caused by the increasing proportion of large-sized precipitates. The decrease in precipitate quantity and the increase in precipitate size led to a decrease in mechanical properties.

## 4. Discussion

### 4.1. Effect of Micro-Alloying Element Zr

During aging strengthening, the addition of nucleation sites can effectively promote and refine the precipitated phases. The maximum solid solution degree of Zr in aluminum is about 0.083%. In this alloy, 0.15% of Zr is added, so part of it is solidly soluble in the aluminum matrix during solidification. The remaining part is precipitated as the primary phase, which provides heterogeneous nucleation sites and refines grains during solidification. For Al-5Cu alloys, after solid solution treatment, the grain size grows. For example, in the Al-5Cu-0.6Mg-0.5Si [21] alloy, after solid solution treatment at 505 °C/2 h, the grain size is about 350~400 μm. In this alloy, due to the addition of Zr, the grain size is 250~300 μm after solid solution treatment at 540 °C/12 h.

The Al_3_Zr phase is not evenly distributed and will be aggregated and distributed in some areas. The resolution temperature of Al_3_Zr is high, and the resolution of the Al_3_Zr phase cannot be achieved at a solid solution (540 °C). There will be secondary precipitation of the Al_3_Zr phase during the heat treatment, which can decrease the energetic barrier for θ′ phase to nucleate and provide nucleation sites [22], making the formed θ′ phase smaller and more diffuse. However, in this paper, due to the small amount of Zr, most of it is precipitated during the non-equilibrium solidification process, and the secondary precipitation is less. After solid solution treatment at 540 °C/12 h, the Al_3_Zr phase was coarsened and the size reached 50–70 μm. It has no effect on hardening.

**Figure 7:**
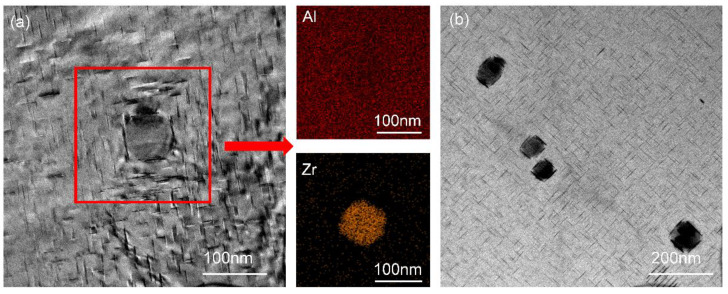
TEM bright-field images of circular-shaped phases: (**a**) one-step aging at 175 °C/16 h; (**b**) two-step aging at 120 °C/4 h + 175 °C/10 h.

No matter whether one uses one-step aging or two-step aging, a circular-shaped phase besides the θ′ phase was found during the precipitation process, as shown in Figure 7. It can be seen that a small number of such circular-shaped phases appear in the alloy treated by one-step peak aging at 175 °C (Figure 7a) and two-step peak aging at 120 °C/4 h + 175 °C/10 h (Figure 7b), and the size is much larger than θ′ phase. It is mainly composed of Al elements and Zr elements. Combined with other studies [23], it can be seen that this is the peritectic reaction between element Zr and Al, and the stable phase with a high melting point Al_3_Zr has been formed. At the same time, according to Figure 7a,b, it can be found that there are a large number of θ′ phases gathered around this Al_3_Zr phase. This is because the Al_3_Zr formed first can be used as the nucleation site of θ′ phase. It shows that the Al3Zr phase can promote the precipitation of θ′ phase.

### 4.2. Effect of Aging Treatment on Precipitation Behavior

The size and distribution of precipitates formed during aging are directly related to the mechanical properties of the alloy. In the low-temperature aging stage of two-step aging, a large number of GP regions can be formed, which can be used as the nucleation site of precipitated phases. Because of the large supersaturation of alloy at low temperatures, the GP regions formed are small and extremely diffuse.

The effects of different aging regimes on the mechanical properties of the Al-Cu-Zr alloy were investigated, and it can be seen that both one-step aging and two-step aging play optimal roles in the mechanical properties of Al-Cu-Zr alloy.

In one-step aging, peak aging at 185 °C has the best mechanical properties. From 155 °C to 185 °C, with the increase of aging temperature, UTS increases by 1.7%, 1.3%, and 2.5%, and YS increases by 1.3%, 0.8%, and 3.3%, respectively. With the increase in temperature, the mechanical properties do not increase significantly.

In the two-step aging process, the alloy with peak aging treatment at 165 °C achieves the best mechanical properties. Compared with the peak aging treatment at 155 °C, the UTS and YS of the alloy are increased by 10.7% and 9.1%, respectively. It can be seen that the effect of aging temperature on mechanical properties in the two-step aging process is far greater than in one-step aging. This is because, with the increase of aging temperature, the quantity of θ′ phases in the alloy increases, and the distribution is more uniform, but the increase of temperature also causes the growth of these precipitates coarsening phenomenon, which is obviously not conducive to the improvement of mechanical properties of the alloy. In the pre-aging stage, a large number of dispersed and stable GP regions appeared, leading to a more dispersed distribution of θ′ phases in the secondary aging step, more precipitates, a larger volume fraction, and no growth coarser phenomenon. Therefore, with the increase in aging temperature, the improvement of mechanical properties of two-step aging is obviously higher than that of one-step aging.

**Figure 8:**
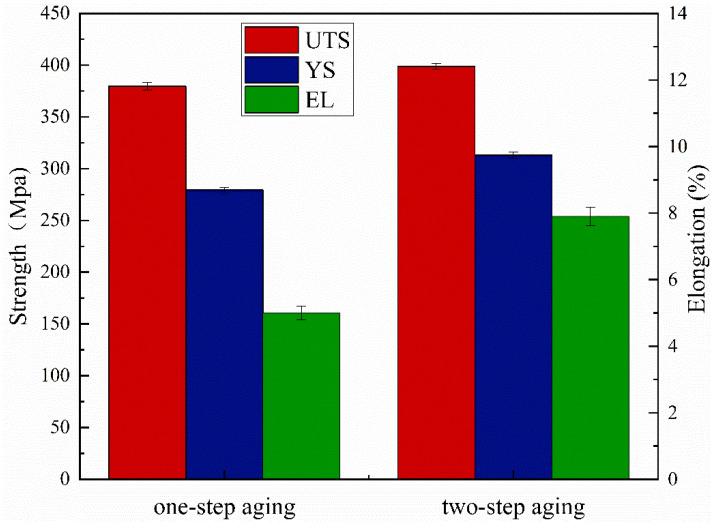
Effect of different aging conditions on the mechanical properties of alloys (one-step aging: 175 °C/16 h; two-step aging: 120 °C/4 h + 175 °C/10 h).

At the same temperature, the pre-aged alloy exhibits better tensile strength, yield strength, and elongation. Figure 8 shows the comparison of mechanical properties after one-step aging at 175 °C/16 h and two-step aging at 120 °C/4 h + 175 °C/10 h. It can be seen that the UTS, YS, and EL of the alloy after two-step aging are increased by 5%, 12%, and 58%, respectively, compared with one-step aging. This is related to the size and distribution of θ′ phases. The comparison between Figure 4b and Figure 6b shows that the number of θ′ phases after pre-aging is significantly greater than that after one-step aging, and the distribution of these θ′ phases is very uniform. Meanwhile, the average length and width of θ′ phases after two-step aging are 31.4 nm and 2.9 nm, respectively. The average length and width of θ′ phases are 35.7 nm and 4.1 nm, respectively, in the alloy without pre-aging treatment. The increased UTS and YS in the two-step aging treatment were attributed to a finer distribution of precipitates. This high precipitate density slows the dislocation movement and thus a higher stress was required for its bowing.

### 4.3. Strengthening Mechanism

In order to better understand the effects of different aging regimes on the mechanical properties of the Al-Cu alloy, the mechanical properties of other previous research results in similar alloy systems are provided in Table 4. As depicted in Table 4, all of these cast alloys exhibit high strengths due to precipitation strengthening. Compared with one-step aging, the mechanical properties of the samples treated by two-step aging are improved to varying degrees. Through the analysis of the microstructure, it was found that the pre-aged alloy obtained a large amount of GP zones precipitation, and the secondary aging step transformed the stable GP zones formed in the pre-aging stage into θ” or θ′ phases, thus greatly enhancing the strengthening effect. The strengthening mechanisms involved in the Al-Cu-Zr alloy include solid solution strengthening, grain size strengthening, dislocation strengthening, etc. The effect of different strengthening mechanisms on the alloy remains to be studied. The overall YS (σ_0_._2_) of Al-Cu alloy can be expressed as:

(1) $$\sigma_{0.2}{=\sigma }_{0}^{\prime }{+\sigma }_{\mathrm{ss}}{+\sigma }_{\mathrm{gs}}{+\sigma }_{\mathrm{or}}$$

where $\sigma_{0}^{\prime }$, $\sigma_{\mathrm{ss}}$, $\sigma_{\mathrm{gs}}$, and $\sigma_{\mathrm{or}}$ represent the yield strength of pure Al, solid solution strength, grain size strengthening, and precipitation strengthening, respectively. It is generally believed that the yield strength of pure Al ($\sigma_{0}^{\prime }$) is about 35 MPa [24], for solid solution strength ($\sigma_{\mathrm{ss}}$), Cu in the Al matrix can provide 7 MPa solid solution strength per mass percent [25], which in this experiment is considered to be 35 MPa, where $\sigma_{\mathrm{gs}}$ is affected by grain size, which is usually calculated by the Hall–Petch equation [26]:

(2) σgs=σ1+kd−12

where $\sigma_{1}$ is the intrinsic resistance of the lattice to dislocation motion (about 29 MPa) [27]. For the constant K, which was reported as 0.17 MPa·m1/2, and d is the average grain size. In this experiment, all samples had similar grain sizes, which by calculating $\sigma_{\mathrm{gs}}$ is about 48 MPa. The Al_3_Zr phase can play a role in refining grains, which is co-latticed with the matrix, effectively improving the thermal stability of the alloy and inhibiting the coarsening of grains during heat treatment. Thus, we can improve the mechanical properties of the alloy.

In this experiment, different aging methods lead to the precipitation of different quantities and sizes of θ′ phases in the alloy. This is the main reason for the difference in mechanical properties, which can be explained by the Orowan equation. Nie and Muddle [28] proposed the modified Orowan equation for the case of strengthening by θ′ phases, this formula describes the relationship between number density, precipitate dimension and thickness, and precipitation strengthening:

(3) $$\sigma_{\mathrm{or}}=\frac{\mathrm{Gb}}{2\pi \sqrt{1-v}}\left(\frac{1}{1.23\frac{1.030}{\sqrt{\mathrm{Nd}}}-\frac{\pi d}{8}-1.061t}\right)\ln\frac{0.981\sqrt{\mathrm{dt}}}{b}$$

where G is the shear modulus of Al (taken as 28 GPa), b is the Burgers’ vector (taken as 0.286 nm), v is Poisson’s ratio (taken as 0.33), and d and t represent the diameter and width of the precipitated phases, respectively. The d of the precipitation phase is 34, 3,7, and 44 nm after 165, 175, and 185 °C one-step peak aging treatments, and the d of the precipitated phase was 28, 34, and 38 nm after two peak aging treatments at 165, 175 and 185 °C. The t of the precipitation phase is 4, 4.1, and 4.5 nm after 165, 175, and 185 °C one-step peak aging treatments, and the t of the precipitated phase was 3.8, 4, and 4.2 nm after two peak aging treatments at 165, 175, and 185 °C. N is the number density, it can be estimated by [28]:

(4) $$N=\frac{4f}{{\pi d}^{2}t}$$

where f is the volume fraction of the θ′ precipitates. The volume fraction of the precipitation phase is 5.5%, 6.5%, and 7% after 165, 175, and 185 °C one-step peak aging treatments, and the volume fraction of the precipitated phase was 8.7%, 7.8%, and 7.7% after two peak aging treatments at 165, 175, and 185 °C. In the peak aging state of one-step aging, the calculated values of the precipitation strengthening are about 150.7, 153.2, and 160.4 MPa for 165, 175, and 185 °C. In the peak aging state of two-step aging, the calculated values of the precipitation strengthening are about 205.3, 189.2, and 182.7 MPa for 165, 175, and 185 °C. It is observed that in the two-step aging stage, the smaller size and higher density of precipitate phases provide a better precipitation-strengthening effect for the alloy. It is consistent with the experimental results.

## 5. Conclusions

In the present study, the effect of different aging regimes on the mechanical properties of Al-Cu-Zr alloy was studied. The main results are summarized as follows:

(1) The Zr added to the alloy forms L_12_-Al_3_Zr during non-equilibrium solidification, which can act as a heterogeneous nucleation site to promote non-uniform nucleation and play a role in refining grains.
(2) The 120 °C/4 h + 165 °C/16 h aging treatment is found to be the optimum two-step aging treatment for the alloy. A large number of stable GP zones are formed in the pre-aging step by two-step aging, and they are transformed into θ′ strengthening precipitate in the secondary aging step. Compared with one-step aging, the θ′ phase is smaller in size and more in quantity, which significantly improves the mechanical properties of the alloy.
(3) The Al_3_Zr phase can be formed in the alloy due to the addition of Zr, which is coherent with the Al matrix. On the one hand, it can promote the precipitation of the θ′ phase and improve the mechanical properties of the alloy; on the other hand, more importantly, the Al_3_Zr phase has the effect of refining the grain and improving the thermal stability of the alloy. Therefore, the addition of Zr can improve the mechanical properties of the alloy in terms of both precipitation strengthening and grain refinement strengthening.

## Figures and Tables

**Table 1:** Chemical composition of the investigated material in wt.%.

	Cu	Mn	Mg	Zr	Er	Ti	Fe	Al
Design	5~5.5	0.3~0.5	0.2~0.3	0.1~0.2	0.1~0.2	<0.2	<0.3	Bal
Actual	5.05	0.35	0.25	0.15	0.14	0.15	0.25	Bal

**Table 2:** Heat treatments applied to the Al-Cu-Zr cast alloy.

Age Type	Solid Solution	Quenching	Pre-Aging	Aging Temperature	Aging Time
One-step aging	540 °C/12 h	Water quenching	-	155–195 °C	0–48 h
Two-step aging	540 °C/12 h	Water quenching	120 °C/4 h	155–195 °C	0–48 h

**Table 3:** Mechanical properties of the Al-Cu-Zr alloy under different aging conditions.

One-Step Aging				Two-Step Aging			
	Ultimate Tensile Strength, MPa	Yield Strength, MPa	Elongation, %		Ultimate Tensile Strength, MPa	Yield Strength, MPa	Elongation, %
As-cast	233.7	173.3	3.2	As-cast	233. 7	173.3	3.2
155 °C/32 h	368.7	273.8	6.7	120 °C/4 h + 155 °C/32 h	373.0	295.7	7.4
165 °C/16 h	375	277.3	5.5	120 °C/4 h + 165 °C/16 h	413.0	322.6	7.6
175 °C/16 h	379.8	279.5	5	120 °C/4 h + 175 °C/10 h	398.7	313.3	7.9
185 °C/6 h	389.3	288.7	4.6	120 °C/4 h + 185 °C/6 h	384.3	307.3	7.8
195 °C/6 h	375.5	275.5	5.7	120 °C/4 h + 195 °C/6 h	374.4	304.6	6.7

**Table 4:** In this paper, the mechanical properties of one and two-step aging in Al-Cu alloys are compared with those published.

	One-Step Aging			Two-Step Aging			Ref
	UTS, MPa	YS, MPa	EL,%	UTS, MPa	YS, MPa	EL,%	
Al-5.5Cu-0.14Zr	379.8	279.5	5	398.7	313.3	7.9	Present study
Al-4.5Cu-3.5Zn-0.5Mg	405.5	341.6	10.9	420.2	368.5	12	[13]
Al-6Cu-0.27Mn	265	120	6.2	305	175	4.5	[29]
Al-5.3Cu-0.8Mg-0.5Ag	505	443	12.2	498	432	16.8	[22]
